# Prim-O-glucosylcimifugin ameliorates DSS-induced ulcerative colitis via suppressing NLRP3 inflammasome activation

**DOI:** 10.3389/fphar.2026.1750305

**Published:** 2026-03-19

**Authors:** Fuqian Wang, Xin Xiong, Qingfeng Ruan, Dan Zhang, Chuanqi Huang, Tingting Xie, Lu Cheng

**Affiliations:** 1 Department of Pharmacy, Wuhan No.1 Hospital, Wuhan, China; 2 School of Foreign Languages, Hubei University of Chinese Medicine, Wuhan, China; 3 Department of Pharmacy, Traditional Chinese and Western Medicine Hospital of Wuhan, Tongji Medical College, Huazhong University of Science and Technology, Wuhan, China

**Keywords:** anti-inflammatory, NLRP3 inflammasome, prim-O-glucosylcimifugin, tight junction protein, ulcerative colitis

## Abstract

**Background:**

Ulcerative colitis (UC) is a chronic and relapsing inflammatory bowel disease (IBD), that lacks specific therapeutic drugs. Prim-O-glucosylcimifugin (POG), a furanochromone glycoside derived from *Saposhnikovia divaricata*, exhibits anti-inflammatory, antioxidant, and anti-inflammasome activities. It has also been shown to inhibit the activation of the NLR family pyrin domain containing 3 (NLRP3) inflammasome. Given that ulcerative colitis (UC) is a chronic inflammatory bowel disease characterized by intestinal barrier dysfunction and NLRP3 activation, this study aims to investigate the therapeutic effects and underlying mechanisms of POG in a murine model of UC induced by dextran sulfate sodium (DSS), and to evaluate its potential translational significance for UC intervention.

**Methods:**

A DSS-induced UC mouse model was employed to evaluate POG’s therapeutic efficacy. Disease activity index, colon length, spleen index, and histopathological alterations of the colonic mucosa were assessed by H&E and AB-PAS staining, transmission electron microscopy, and immunofluorescence. Proinflammatory cytokines (TNF-α, IL-6, IL-18, IL-1β) were measured by ELISA, and NLRP3 inflammasome–related proteins were analyzed by Western blot.

**Results:**

POG treatment alleviated weight loss, colon shortening, splenomegaly, and inflammatory infiltration. It also restored goblet cell mucin droplets and tight junction proteins (Occludin, ZO-1, Claudin-1, MUC2), and reduced villi exfoliation. Serum levels of TNF-α, IL-6, IL-1β, and IL-18 were decreased. Western blot and ELISA revealed that POG suppressed NLRP3 inflammasome formation, inhibited GSDMD activation, and reduced cleavage and release of IL-1β and IL-18. Collectively, these findings indicate that POG not only attenuates inflammatory injury but also protects epithelial barrier structure and function, supporting its potential value in mechanism-based UC therapy development.

**Conclusion:**

POG ameliorates experimental UC by inhibiting NLRP3 inflammasome activation, thereby mitigating colonic inflammation, preserving mucosal barrier integrity, and blocking downstream inflammatory responses. POG shows promise as a therapeutic agent for UC and provides a rationale for further preclinical optimization and future clinical translation.

## Graphical Abstract

**Figure:**
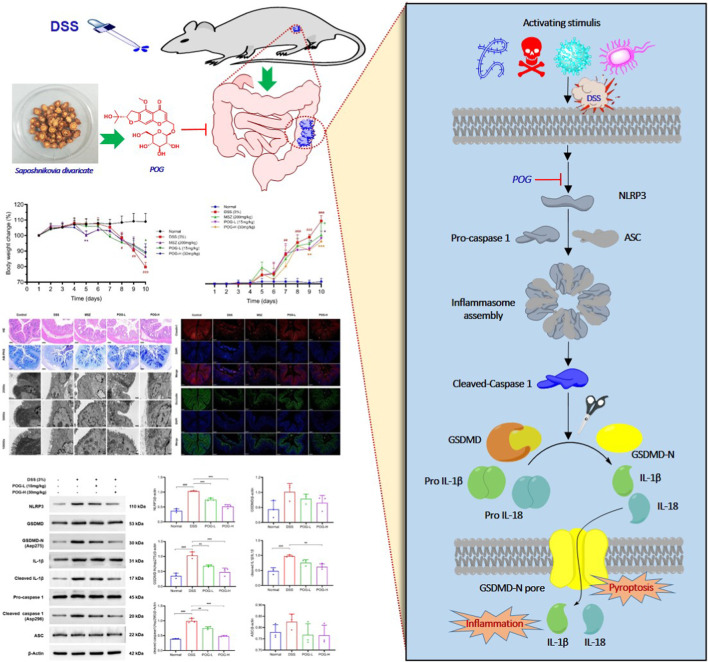

## Highlights

- POG treatment ameliorates DSS-induced ulcerative colitis.
- POG attenuates intestinal barrier damage and suppresses the cleavage and release of IL-1β and IL-18.
- POG suppresses the NLRP3 inflammasome/GSDMD pathway, thereby ameliorating intestinal barrier function in UC mice.

## 1 Introduction

Ulcerative colitis (UC) is a chronic inflammatory disease characterized by recurrent and persistent inflammation occurring between the colon and rectum, presenting with clinical features such as abdominal pain, diarrhea, hematochezia and weight loss (Chen et al., 2020; Zhong et al., 2022). With changes in dietary habits and improvement of living standards, UC, which is prevalent in Western populations, is gradually becoming a globally challenging disease (Du and Ha, 2020). Owing to the involvement of the entire colon and terminal ileum in UC, the onset is often insidious with intermittent relapses, achieving a complete cure is challenging. This situation increases the risk of disease progression, facilitating the potential transition of colitis into colorectal cancer, which significantly compromises the quality of life for affected individuals (Olén et al., 2020). The etiology of UC is multifaceted and remains incompletely understood. Individuals with genetic predispositions are influenced by environmental factors, leading to alterations in gut microbiota, immune dysregulation and defects in the intestinal mucosal barrier, all of which are thought to be intricately linked to the pathophysiology of UC (Goodman et al., 2020). The NLR family pyrin domain containing 3 (NLRP3) inflammasome plays a critical role in the regulation of various signaling pathways involved in immunological and inflammation-related diseases. Its activation initiates the processing of pro-inflammatory cytokines and releases the inflammatory cytokines interleukin (IL)-1β and IL-18, concomitantly cleaving gasdermin D (GSDMD), subsequently mediating pyroptosis, initiating downstream inflammatory responses and promoting disease progression. NLRP3 inflammasome represents a potential therapeutic target for anti-inflammatory (Coll et al., 2022). Its involvement has been linked to the pathogenesis of inflammatory bowel disease (IBD), which encompass Crohn’s disease and UC (Mangan et al., 2018). Suppression of inflammasome activation has been shown to ameliorate colitis (Bauer et al., 2010).

Currently, 5-aminosalicylic acid (5-ASA), immunosuppressants and biologics are commonly used in clinical practice for the treatment of UC. However, these medications are associated with unavoidable side effects such as drug resistance and frequent relapses (Wang et al., 2024). In particular, long-term use of certain agents, especially corticosteroids, may cause adverse effects such as insomnia, hypertension, and osteoporosis. Furthermore, the recurrence of disease may necessitate surgical resection of the colon, which carries additional risk of complications (Sharma et al., 2023; Zhao et al., 2023). Therefore, it is imperative to explore the mechanisms underlying the alleviation or suppression of UC and to identify new active compounds that are safe, effective and viable as alternative therapeutic targets for UC.

**FIGURE 1:**
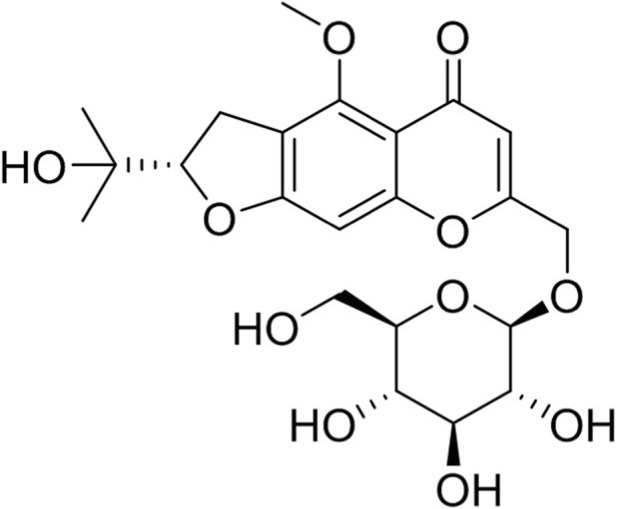
Structure of Prim-O-glucosylcimifugin (POG).

Pharmacologically active constituents of traditional medicinal herbs and natural medicinal plants, informed by their traditional applications, are integral to contemporary medical research and practice. In recent years, the isolation and identification of monomer compounds with specific pharmacological activities from traditional medicinal herbs have provided validated molecular entities for drug screening and discovery (Newman and Cragg, 2020). For instance, 6-gingerol, Oroxindin and Dihydroberberine have shown promising effects in the treatment of UC (Li et al., 2021; Li et al., 2024; Liu, Q. et al., 2020). Drawing from a comprehensive review of the literature and research on traditional herbal plants, we performed an analysis and screening of compounds derived from these herbal treatments. According to Traditional Chinese Medicine (TCM), *Saposhnikovia divaricata* (Fangfeng) possesses the efficacy of “expelling dampness and alleviating pain”. As documented in classical Chinese medical texts such as “*Danxi’s Mastery of Medicine*”, formulations containing *S. divaricata*, such as “*Tongxie Yaofang*” are primarily indicated for intestinal borborygmi, abdominal pain, and diarrhea, and are used in the treatment of acute enteritis and chronic colitis. Additionally, “*Fangfeng Siling San*” demonstrated spleen-strengthening, dampness-draining, and diarrhea-stopping effects. It is effectively used to treat loose stools and diarrhea associated with dampness and spleen deficiency. In subsequent validation experiments, we observed that Prim-O-glucosylcimifugin (POG), Anemoside A3, resveratrol and piperine demonstrated different levels of disease symptoms mitigations in dextran sulfate sodium (DSS)-induced UC mice. POG (Figure 1), a major component of traditional Chinese medicine (TCM) for *Saposhnikovia divaricate*, is also found in a few other herbal materials, which have been shown in modern pharmacological studies to possess anti-inflammatory and anti-tumor biological activity effects, while also exhibiting inflammasome inhibitory activity (Gao et al., 2019; Liu et al., 2022). However, the specific mechanism of action of POG in the treatment of UC remains unclear. Therefore, this study aimed to examine the therapeutic efficacy of POG in UC, to clarify its potential mechanisms in a DSS-induced UC model, and to provide insights for the development of UC-targeted therapeutic agents.

## 2 Materials and methods

### 2.1 Drugs and reagents

POG (C_22_H_28_O_11_; molecular weight: 468.45; batch number: MUST-23041910) was procured from Manst (Chengdu) Biotechnology Co., Ltd. (Chengdu, China). DSS (molecular weight: 36,000–50000) was purchased from MP Biochemicals (OH, United States). Transmission electron microscopy (TEM) fixative (P1126) was purchased from Beijing Solaibao Technology Co., Ltd. (Beijing, China). Phosphate buffered saline (PBS, AS1044) and Ethylene Diamine Tetraacetic Acid (EDTA, S10014) antigen retrieval buffer (pH 8.0) were purchased from Siwega Biotech (Wuhan) Co., Ltd. (Hubei, China), bovine serum albumin (BAS, 10,711,454,001) and 4,6-Diamidino-2-phenylindole (DAPI, D9542) were purchased from Sigma Chemical Co. (MO, United States). RIPA lysis buffer (P0013D) was supplied by Beyotime Biotech Inc (Shanghai, China), polyvinylidene fluoride (PVDF, IPVH00010) membranes were acquired from Merck Millipore (Boston, MA, United States), and Tween 20 (T8220) was purchased from Beijing Solaibao Technology Co., Ltd. (Beijing, China). Hematoxylin and eosin (H&E, G1120) and Alcian blue and periodic acid-Schiff (AB-PAS, G1482) staining kits were procured from Siwega Biotech (Wuhan) Co., Ltd. (Hubei, China). Enzyme-linked immunosorbent assay (ELISA) kits including IL-1β (RX203063M), tumor necrosis factor (TNF)-α (#RX202412M), IL-6 (#RX203049M) and IL-18 (#RX203064M) were purchased from Quanzhou Ruixin Biotechnology Co., Ltd. (Fujian, China). Pierce™ Rapid Gold BCA Protein Assay Kit (#A53225) was procured from Thermo Fisher Scientific. Antibodies of zonula occludens-1 (ZO-1, 21773-1-AP), Occludin (66378-1-Ig), Claudin-1 (#13050-1-AP), Mucin 2 (MUC2, 27675-1-AP), NLRP3 (30109-1-AP) and GSDMD (20770-1-AP) were purchased from Sanying Biotech (Wuhan) Co., Ltd. (Hubei, China), GSDMD-N(Asp275, 36,425), Cleaved IL1β (63,124) and Cleaved caspase1(Asp296, 89,332) antibodies were purchased from Cell Signaling Technology (Danvers, MA, United States), β-Actin (ab8226), IL1β (A22257), pro-caspase1 (A0964) and apoptosis-associated Speck-like protein Containing CARD (ASC, A22046) antibodies were purchased from Abclonal, Cy3-labeled goat anti-rabbit IgG H + L (111–165-003) and 488-labeled goat anti-rabbit IgG H + L (111–545-003) applied for immunofluorescence (IF) were from Jackson ImmunoResearch Inc. (PA, United States).

### 2.2 Mice

Eight-week- old male C57BL/6 mice were purchased from Hunan SJA Laboratory Animal Co., Ltd., and implementation standards of Huazhong University of Science and Technology official review of experimental animal ethics, Wuhan, China (IACUC number: 3,793) were strictly required throughout the experiment. All mice were provided with *ad libitum* access to food and purified water, housed under a 12 h light/dark cycle, maintained at relative humidity of 60% ± 5%, and a constant temperature ranging from 20 °C to 22 °C in a specific pathogen-free (SPF) environmen.

**FIGURE 2:**
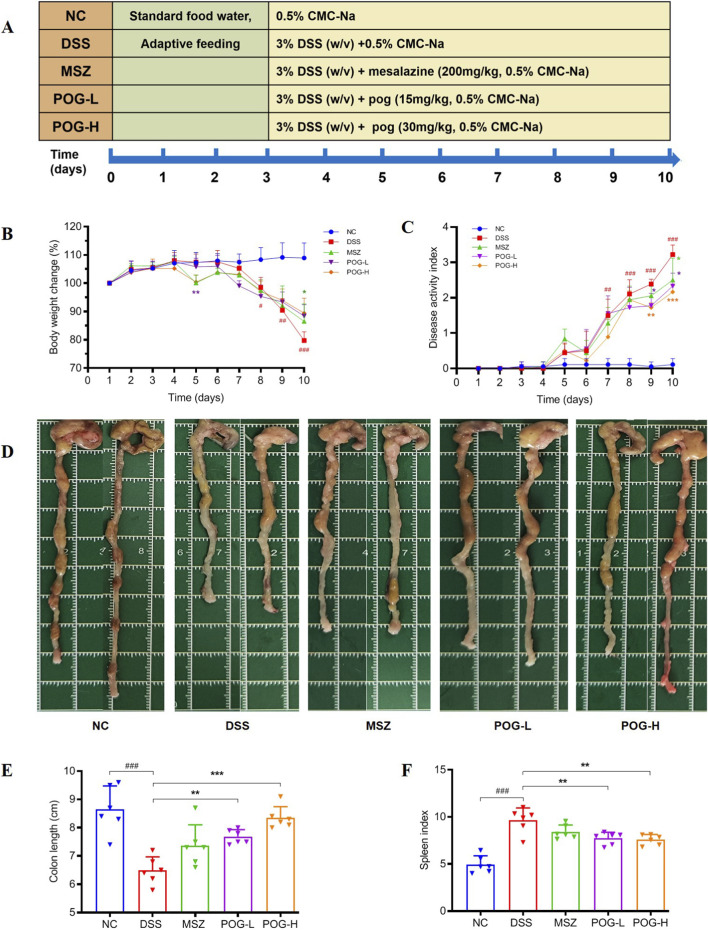
Effects of POG on physiological parameters in DSS-induced UC mice. **(A)** Schematic summary of animal experiment; **(B)** Body weight change; **(C)** Disease activity index (DAI); **(D)** Representative macroscopic view of colon represented in each group mice; **(E)** Length statistics of the colon in each group mice (n = 6); **(F)** Spleen index in each group of mice (n = 6). Data are presented as mean ± SEM (n = 6). NC: normal control; DSS: DSS-induced UC mice; MSZ: DSS-induced UC mice + MSZ gavaged (200 mg/kg); POG-L: DSS-induced UC mice + POG-L (15 mg/kg); POG-H: DSS-induced UC mice + POG-H (30 mg/kg); ^#^ *P* < 0.05, ^##^ *P* < 0.01 and ^###^ *P* < 0.001vs NC group; ^*^ *P* < 0.05, ^**^ *P* < 0.01 and ^***^ *P* < 0.001vs DSS group.

The complete experimental process is shown in Figure 2A, mice were randomly divided into 5 groups (n = 6), including normal control (NC) group, DSS group, mesalazine (MSZ, 200 mg/kg) group, POG-L group (15 mg/kg) and POG-H group (30 mg/kg) on the first day of after 3 days of acclimatization. The dosage of MSZ was calculated by converting the body surface area of mice and humans in accordance with the clinical dosage and commonly adopted doses in the literature (Lv et al., 2021; Li et al., 2015), and the dosage of POG were appropriately modified based on the literature review and previous studies (Cheng et al., 2023; Wang et al., 2023; Yu et al., 2024).

Except for NC group which drank purified water, the other four groups all drank 3% DSS (diluted in purified water, freshly prepared every other day) in the daily intake of water. NC and DSS groups were given 0.3% CMC-Na (a solvent used to dissolve MSZ and POG) by gavage, MSZ and POG groups were intragastrical administered the 5-aminosalicylic acid (200 mg/kg) and POG (15 and 30 mg/kg) daily, which the above procedures were performed once a day (0.15 mL/10 g) for 7 consecutive days. On the 11th day of the experiment, mice were anesthetized with phenobarbital, and blood samples were collected via the retro-orbital vein under anesthesia, followed by cervical dislocation and collection of colon tissues. Histopathological samples were stored in different reagents, and the remaining tissues were stored at −80 °C. Plasma samples were left at 4 °C overnight and centrifuged to obtain serum for subsequent analysis.

### 2.3 Disease activity index (DAI)

The mice weight, hardness of feces and fecal occult blood (using fecal occult blood test kits, Solarbio, BC8270) were recorded daily during the experimental period. DAI were calculated using the well-established system (Lu et al., 2023), as follows: DAI = [weight loss rate score (0, <1%; 1, 1%–5%; 2, 5%–10%; 3, 10%–15%; 4, ≥15%) + stool hardness score (0, normal; 1, soft stools; 2, mucoid stools; 3, loose stools) + stool blood score (0, negative; 2, positive; 4, gross bleeding)] divided by three in each mouse (Table 1). “Soft stool” refers to unformed, paste-like stools that are softer than normal fecal pellets but not watery, while “mucoid stool” denotes stools with visible mucus coating or mucus mixed throughout the fecal matter.

**TABLE 1:** DAI scoring criteria.

Score	Weight loss %	Stool consistency	Blood in stool
0	<1	Normal	Negative
1	1–5	Soft	​
2	5–10	Mucoid	Positive
3	10–15	Diarrhea	​
4	≥15	​	Gross bleeding

### 2.4 Histological examination

Colon tissues were fixed in 10% neutral formalin solution more than 24 h and then embedded in paraffin, cut into thickness slice, then deparaffinized and hydrated sequentially in xylene and graded ethanol, respectively. Tissue sections were stained with H&E and AB-PAS, followed by examination under a light microscope for histopathological analysis to capture tissue morphological changes, inflammatory cell infiltration and destruction of the intestinal mucus layer were observed and recorded (Pannoramic, 3DHISTECH, Hungary) (Qian et al., 2023; Wei, Y. et al., 2021). In H&E staining, nuclei were stained blue and the cytoplasm was stained red. In AB-PAS staining, acid mucin stains blue, and mixed mucus, composed of glycogen and mucin, stains blue-purple.

### 2.5 Evaluation of transmission electron microscopy (TEM)

The colonic tissue specimens were immersed in TEM fixative (2.5% glutaraldehyde) at 4 °C for 2 h, followed by fixation in 1% (w/v) osmium tetroxide-0.1M PBS for 2 h at room temperature. Colon samples were dehydrated in low-to-high concentrations of ethanol and embedded in 812 resin. Slices with 60–80 nm thick were mounted with copper mesh and stained with 2.6% uranyl acetate and lead citrate. All sections were imaged using TEM (JEM-1400 Flash, JEOL, Japan) for observation and analysis.

### 2.6 Enzyme-linked immunosorbent assay (ELISA)

Blood samples obtained from anesthetized mice were collected overnight at 4 °C, centrifuged (13,000 rpm for 20 min) to obtain serum and stored at −80 °C for inflammatory factors analysis. The levels of IL-1β, TNF-α, IL-6 and IL-18 were measured by ELISA kits according to the manufacturer’s instructions.

### 2.7 Immunofluorescence (IF)

Paraffin embedded colon tissue sections were dewaxed and hydrated with xylene and graded ethanol solutions, respectively. Then the sections were placed in a repair box filled with EDTA antigen repair buffer (pH 8.0) in a microwave oven for antigen repair. The tissue was circled with a histochemical pen to prevent antibody from flowing away after the sections were slightly dry, BAS was then added dripping and incubated for 30 min. Subsequently, the sections were stained with a certain ratio of primary antibody and incubated overnight at 4 °C, followed by washing with PBS three times and incubation with the secondary antibody at room temperature for 50 min keep in dark place. Slices were washed with PBS for three times again, followed by adding DAPI, sealed with an anti-fluorescence quenching agent, and observed under a fluorescence microscope to capture images (DM2000LED, LEICA; Pannoramic MIDI, 3DHISTECH, Hungary). The fluorescence gray value was determined by ImageJ 1.54 g.

### 2.8 Western blotting (WB)

The colon tissues were rinsed with pre-cooled PBS buffer to remove blood contaminants, cut into small pieces, mixed with RIPA tissue lysis buffer, placed in an ice bath, homogenized, and then centrifuged to extract the total protein solution and denature the protein solution to obtain tissue samples. Purified tissue samples were separated using sodium dodecyl sulfate-polyacrylamide gel electrophoresis and transferred to PVDF membranes. Following the addition of the primary antibodies (NLRP3, GSDMD, GSDMD-N, IL-1β, Cleaced IL-1β, pro-caspase 1, Cleaced caspase 1 and ASC), the membranes were incubated overnight at 4 °C for blocking, and then washed five times with Tris Buffered Saline Tween (TBST, 0.1%, v/v), and each wash lasted 5 min. Subsequently, the secondary antibody and horseradish peroxidase were added, and the membranes were incubated at 37 °C for 1 h. Chemiluminescent substrate was evenly applied to the protein side of PVDF membranes after washing with TBST. Following the reaction, luminescence detection was performed and images were captured for archival analysis (Monad, Suzhou, China).

### 2.9 Statistical analysis

GraphPad Prism 8.0 software (GraphPad Software, Inc., CA, United States) was used for perform and plotting the experiments data. All experimental results are presented as mean ± standard error of measurement (SEM) and one-way analysis of variance (ANOVA) followed by Tukey’s test were implemented. The statistical significance level was expressed *p* < 0.05.

## 3 Results

### 3.1 POG relieved DSS-induced UC symptoms in mice

Mice with DSS-induced UC exhibit significant weight loss, diarrhea and hematochezia symptoms (Chassaing et al., 2014). To investigate the alleviating effect of POG on the symptoms of UC in mice, the experimental process depicted in Figure 2A was meticulously implemented, and recorded of mice weight, fecal characteristics and rectal bleeding on each day during this period. In Figure 2B, it is evident that percentage change in body weight (Body weight change was calculated as the weight on each day relative to the initial weight, expressed as a percentage.) of DSS group significantly decreased compared to NC group starting from day 8 of the experiment (*P* < 0.05). However, on day 10, POG-H showed a trend toward preventing weight loss, with POG-L having a significant but transient effect on a single day (*P* < 0.05). The DAI score, which is a composite measure, provides stronger evidence of efficacy (Figure 2C, POG-L, *P* < 0.05; POG-H, *P* < 0.001). Furthermore, at the specified dosage, colonic shortening (Figures 2D,E, POG-L, *P* < 0.01; POG -H, *P* < 0.001) and splenomegaly caused by the inflammatory response (Figure 2F, POG -L and H, *P* < 0.01) in DSS-induced UC mice were attenuated compared to DSS groups.

### 3.2 POG improved intestinal shielding in UC mice

**FIGURE 3:**
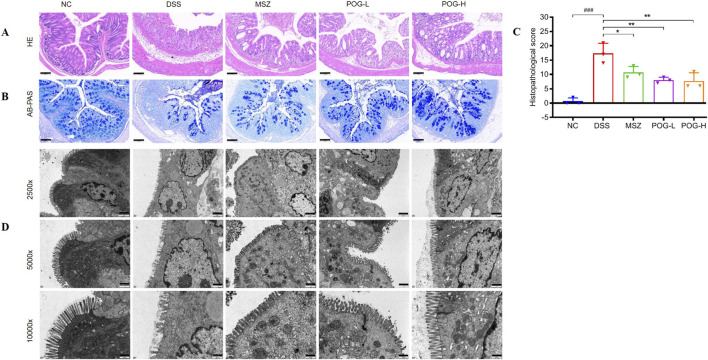
Therapeutic effects of POG on histopathology of colon tissue in DSS-induced UC mice. **(A)** Histopathological damage of colons was detected by H&E and **(B)** AB-PAS staining in each group mice (magnification ×15, scale bar = 100 μm); **(C)** Histopathological scores were determined for the colon tissue samples. ^###^ *P* < 0.001 vs. NC group; ^*^ *P* < 0.05 and ^**^ *P* < 0.01vs DSS group; **(D)** TEM images of distal colon epithelial ultrastructure and morphology in each group mice (magnification 2,500, 5,000 ×, and 10,000 ×, scale bar = 2, 1 *μ*m, and 500 nm).

The results of H&E staining indicated that colonic mucosa in NC group of mice was intact, free from inflammatory cell infiltration and edema, with clear crypt morphology, goblet cell morphology was normal and evenly distributed. Whereas, exposure to DSS resulted in hypertrophy of the muscularis layer, accompanied by epithelial crypts loss or structural distortion, characterized by branching and twisting crypts. In POG groups, there was a reduction in inflammatory cell infiltration, decreased crypt damage, and restoration of ileal wall integrity compared to DSS group (Figure 3A). Histopathological scores (Figure 3C) were determined according to the criteria described in the literature (Wang et al., 2017). AB-PAS staining revealed disruption of the intestinal mucus barrier, as evidenced by reduced thickness in DSS-induced colitis mice. POG administration stimulated mucus secretion, leading to increased intestinal mucus layer thickness compared to DSS group (Figure 3B).

The TEM (Figure 3D) revealed that microvilli on surface of ileum in the NC group were dense, regularly arranged, and exhibited tight intercellular connections. In the DSS group, intestinal microvilli were fractured and incomplete, sparse, and varied in length. The POG-L group exhibited improved microvilli morphology, with slight shortening and reduced intercellular space expansion compared to DSS group. In the POG-H group, epithelial cells appeared near-normal, displaying dense, well-ordered microvilli.

### 3.3 POG improves colonic permeability in UC mice by regulating tight junction and mucin 2 proteins

**FIGURE 4:**
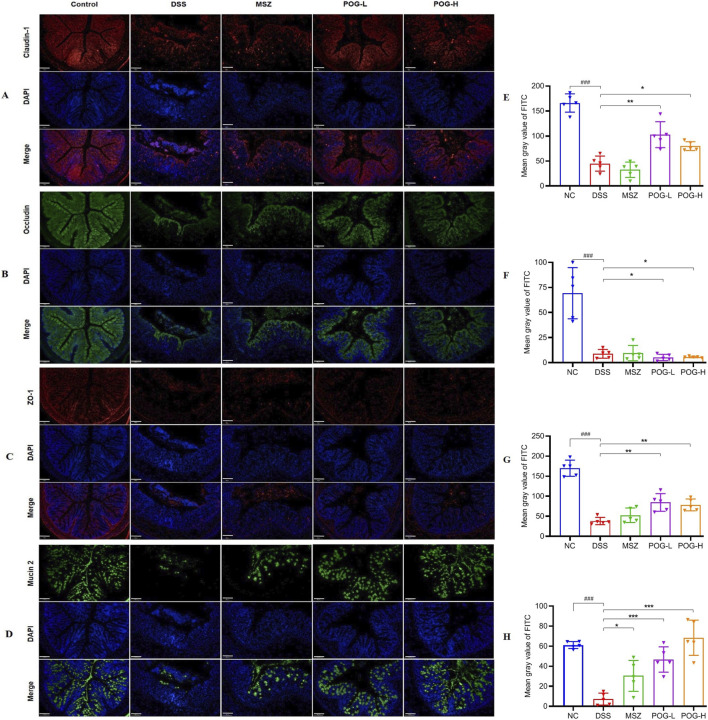
Therapeutic effects of POG on colon immune barrier protein expression in DSS-induced UC mice. Representative immunofluorescence staining for Claudin-1 (**(A)**, red), Occludin (**(B)**, green), ZO-1 (**(C)**, red) and Mucin 2 (**(D)**, green) with DAPI (blue). (magnification ×15, scale bar = 100 *μ*m); Mean gray value of Claudin-1, Occludin, ZO-1 and Mucin 2 **(E–H)**; ^###^ *P* < 0.001 vs. NC group; ^*^ *P* < 0.05, ^**^ *P* < 0.01 and ^***^ *P* < 0.001 vs. DSS group.

The integrity of the intestinal epithelium, maintained primarily by tight junction (TJ) proteins, is essential for preventing inflammation and preserving internal homeostasis (Neurath et al., 2025; Peng et al., 2024; Zhu et al., 2024). Immunofluorescence of TJ proteins (Claudin-1, Occludin and ZO-1) showed a continuous, circumferential localization along the epithelial border in NC mice, whereas DSS induced a marked loss of this continuous staining with a fragmented, discontinuous pattern and reduced signal (Figures 4A–C). POG treatment largely restored the continuous distribution of these TJ proteins at the epithelial border, contrasting with the fragmented pattern in the DSS group, and quantitative fluorescence analysis confirmed partial recovery of protein levels (Figures 4E–H). MUC2 produced by goblet cells is a key component of the mucus layer protecting the epithelium (Kang et al., 2022; Liu, Y. et al., 2020), and DSS markedly reduced MUC2 expression and mucus thickness (Jiang et al., 2022). POG markedly enhanced MUC2 staining and increased the thickness of the mucus layer, with effects greater than those observed in the MSZ group (Figure 4D). Taken together, these findings indicate that POG repairs DSS-induced colonic mucosal barrier damage by restoring the continuous localization of TJ proteins and by rescuing goblet-cell mucin secretion.

### 3.4 POG decreased inflammatory cytokines production in DSS-induced UC mice

**FIGURE 5:**
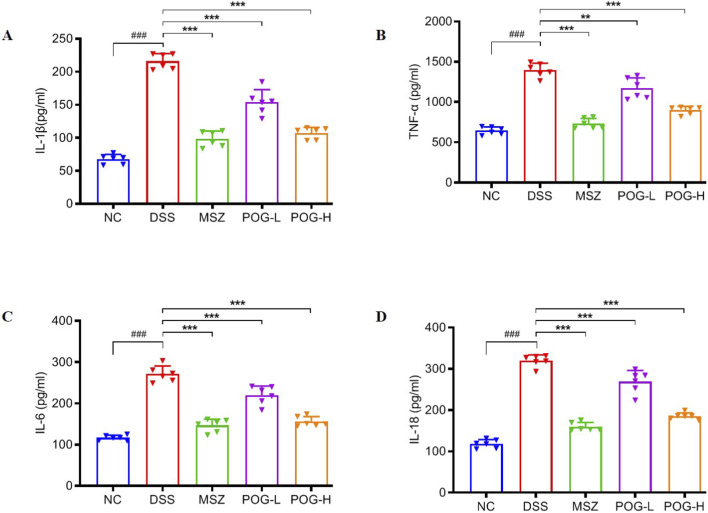
Effect of POG on levels of IL-1β **(A)**, TNF-α **(B)**, IL-6 **(C)** and IL-18 **(D)**. Data are presented as mean ± SEM (n = 6). NC: normal control; DSS: DSS-induced UC mice; MSZ: DSS-induced UC mice + MSZ gavaged (200 mg/kg); POG-L: DSS-induced UC mice + POG-L (15 mg/kg); POG-H: DSS-induced UC mice + POG-H (30 mg/kg); ^###^ *P* < 0.001 vs. NC group; ^**^ *P* < 0.01 and ^***^ *P* < 0.001 vs. DSS group.

The abnormal expression levels of inflammatory factors in mice are closely associated with the severity of UC and represent one of its significant characteristics. ELISA results indicated that levels of serum inflammatory factors IL-1β, TNF-α, IL-6 and IL-18 in DSS groups are significantly increased compared with NC groups (*P* < 0.001). In addition to POG-L group in TNF-α showed a less significant reduction compared with DSS group (*P* < 0.01), the remaining POG-L and POG-H groups of all cytokines showed particularly significant reductions compared with DSS groups (*P* < 0.001) (Figure 5).

### 3.5 POG inhibited NLRP3 inflammasome activation in DSS-induced UC mice

The NLRP3 inflammasome is an innate immune receptor associated with various inflammatory diseases in humans, such as diabetic nephropathy, non-alcoholic steatohepatitis and diabetes (Deng et al., 2022; Ding et al., 2018; Wang et al., 2018; Zhang et al., 2022). As a central component of inflammasome signaling, NLRP3 expression substantially influences inflammatory responses. Activation of the NLRP3 inflammasome promotes the maturation and release of IL-1β and IL-18, concurrent with the onset of inflammatory pyroptosis via GSDMD pore formation, which exacerbates inflammatory responses (Swanson et al., 2019). Accumulating experimental evidence highlights the pathogenic significance of NLRP3 inflammasome activation in UC (Błażejewski et al., 2017; Lv et al., 2021), making its blockade an attractive therapeutic intervention for this inflammatory bowel disease.

**FIGURE 6:**
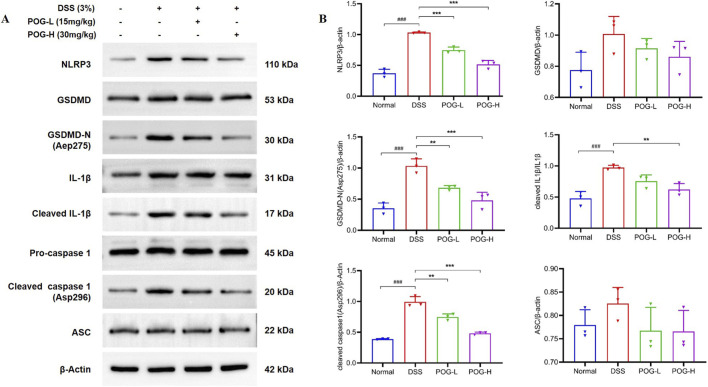
**(A)** Effect of POG on NLRP3 and IL-1β in UC mice by Western blot (expression of protein bands on NLRP3, GSDMD, GSDMD-N, IL-1β, Cleaced IL-1β, Pro-caspase 1, Cleaced Caspase 1 and ASC. **(B)** Quantitative analysis of NLRP3, GSDMD, GSDMD-N, Cleaced IL-1β/IL-1β, Cleaced Caspase 1 and ASC). Data are presented as mean ± SEM (n = 3). NC: normal control; DSS: DSS-induced UC mice; MSZ: DSS-induced UC mice + MSZ gavaged (200 mg/kg); POG-L: DSS-induced UC mice + POG-L (15 mg/kg); POG-H: DSS-induced UC mice + POG-H (30 mg/kg); ^###^ *P* < 0.001 vs. NC group; ^**^ *P* < 0.01 and ^***^ *P* < 0.001 vs. DSS group.

To clarify whether the protective effect of POG was associated with inhibition of NLRP3 inflammasome activation, we conducted *in vivo* experiments, and the effects of POG on NLRP3 inflammasome were presented in Figure 6. The Western blotting analysis showed that the proteins of NLRP3/β-actin, GSDMD-N/β-actin, cleaced IL-1β/IL-1β and cleaced caspase 1/β-actin were dose-negatively correlated with POG. As shown in Figure 6, the levels of protein bands of NLRP3/β-actin (*P* < 0.001), GSDMD-N/β-actin (*P* < 0.001), Cleaced IL-1β/IL-1β (*P* < 0.001) and Cleaced Caspase 1/β-actin (*P* < 0.001) in DSS groups were remarkably increased by 1.77-fold, 1.94-fold, 1.03-fold and 1.55-fold compared to NC groups, respectively. The data presented indicate that NLRP3 are activated during the pathogenesis of UC. Subsequently, POG was administrated, and the expression of gray-scale value in NLRP3/β-actin (*P* < 0.001), GSDMD-N/β-actin (*P* < 0.001), Cleaced IL-1β/IL-1β (*P* < 0.01) and Cleaced Caspase 1/β-actin (*P* < 0.001) were inhibited in POG-H groups when compared with DSS groups, and the expression of protein significantly downregulated by 50%, 54%, 36% and 51%, respectively. The aforementioned results suggest that POG may ameliorate DSS-induced UC by suppressing inflammasome activation, thereby reducing the activation and release of inflammatory mediators such as IL-1β, and inhibiting pyroptosis.

## 4 Discussion

Ulcerative colitis (UC), as a chronic inflammatory bowel disease, is characterized by typical pathological features, including disruption of the intestinal mucosal barrier, colonic inflammatory infiltration and clinical manifestations such as diarrhea, mucous and bloody stools, and colonic shortening (Li et al., 2023). As the disease progresses, persistent intestinal ulcers significantly elevate the risk of colorectal cancer, rendering UC a major global health concern (Xiao et al., 2022). Current research indicates that the pathogenesis of UC involves a complex interplay of impaired intestinal barrier function, autoimmune dysregulation and environmental factors. Although commonly used therapeutic agents such as 5-aminosalicylic acids, immunosuppressants and TNF-α antagonists demonstrate certain efficacy, their long-term use may lead to adverse effects including gastrointestinal reactions, bone marrow suppression and even tumorigenesis (Fernández-Clotet et al., 2019; Miao et al., 2021). Notably, restoring the damaged intestinal mucosal barrier has been identified as a critical therapeutic target in UC management. In recent years, TCM and its active constituents have shown unique advantages in UC treatment due to their proven efficacy and minimal adverse reactions, providing a promising avenue for the development of novel anti-UC drugs (Guazelli et al., 2021).

This study employed a DSS-induced UC mice model, which effectively replicates key histopathological features of human UC, including weight loss, hematochezia and colonic shortening. The experimental system evaluated the therapeutic efficacy of different doses of POG (15 mg/kg and 30 mg/kg). Results demonstrated that POG-L significantly ameliorated weight loss in UC mice (*P* < 0.05), diarrhea and hematochezia (POG-L, *P* < 0.05; POG-H, *P* < 0.001), while also effectively alleviating pathological changes such as increased spleen index (POG-L, *P* < 0.01; POG-H, *P* < 0.01) and colonic shortening (POG-L, *P* < 0.01; POG-H, *P* < 0.001) (Figure 2). Histological analysis further confirmed that, compared to DSS model group, POG-treated groups exhibited a marked reduction in inflammatory cell infiltration within the colonic mucosa, improved epithelial integrity, and significant alleviation of pathological alterations such as crypt disorganization and goblet cell depletion (Figure 3). These findings suggest that POG mitigates DSS-induced morphological damage in colonic tissue by preserving the integrity of the intestinal mucosal barrier, thereby exerting a therapeutic effect in alleviating UC.

Tight junction (TJ) protein complexes, including claudin-1, occluding, ZO-1, and MUC2, constitute the core components of the intestinal physical barrier. The integrity of the TJ structure effectively seals intercellular gaps, thereby preventing the invasion of external harmful substances and maintaining the physiological function and integrity of the intestinal barrier (Wang et al., 2019). DSS induction results in a reduction of goblet cell numbers, leading to decreased mucin secretion, downregulation of TJ proteins (claudin, occludin and ZO-1), and ultrastructural alterations such as sparse and atrophic microvilli. Notably, POG treatment significantly upregulates the expression of TJ proteins and MUC2 (Figures 3, 4), reconstructing the fundamental framework of the intestinal mucus layer and enhancing barrier function. Electron microscopy reveals that POG reduces DSS-induced villus shedding and restores the morphological integrity of the intestinal mucosa. These findings demonstrate that POG exerts therapeutic effects on DSS-induced colitis in mice by restoring TJ barrier integrity and promoting mucosal healing, thus providing morphological evidence for its mechanism in repairing intestinal barrier function.

Our experiments demonstrated that POG significantly suppressed the expression of NLRP3, GSDMD-N, Cleaced IL-1β and cleaced caspase 1 (Figure 6), consistent with the observed decline in pro-inflammatory cytokines such as TNF-α, IL-6, and IL-18 detected by ELISA. These findings suggest that POG may exert its therapeutic effect in UC by inhibiting NLRP3 inflammasome activation, thereby attenuating downstream inflammatory cascades and alleviating disease pathology. Existing evidence indicates that POG, as an active component of TCM, exerts anti-ulcerative colitis effects through suppression of inflammatory pathways including MAPK, AKT, and NF-κB (Yin et al., 2022). It has been confirmed that the NLRP3 inflammasome plays a pivotal role in UC activity and is associated with the secretion of inflammatory cells; blockade of NLRP3 inflammasome activation has been shown to mitigate UC symptoms (Li et al., 2022). To further elucidate the mechanistic role of POG in UC, we investigated its impact on NLRP3 inflammasome activation.

The NLRP3 inflammasome (NLRP3/ASC/pro-caspase-1) activates caspase-1 to generate cleaved caspase-1, driving maturation of IL-1β/IL-18 and GSDMD dependent pyroptosis, thereby amplifying inflammation and compromising barrier integrity (Fang et al., 2024; Fu et al., 2021; Wei, Y. Y. et al., 2021; Yan et al., 2020; Zhu et al., 2021). Concurrently, excessive expression of TNF-α, a key driver of intestinal inflammation, could induce endothelial cell apoptosis, further compromising intestinal barrier integrity (Friedrich et al., 2019). In our study, the results indicate that POG protects the colonic mucosal barrier at least in part by inhibiting the NLRP3/caspase-1/IL-1β axis and downstream pyroptotic/inflammatory signaling, thereby reducing epithelial damage and inflammation.

**FIGURE 7:**
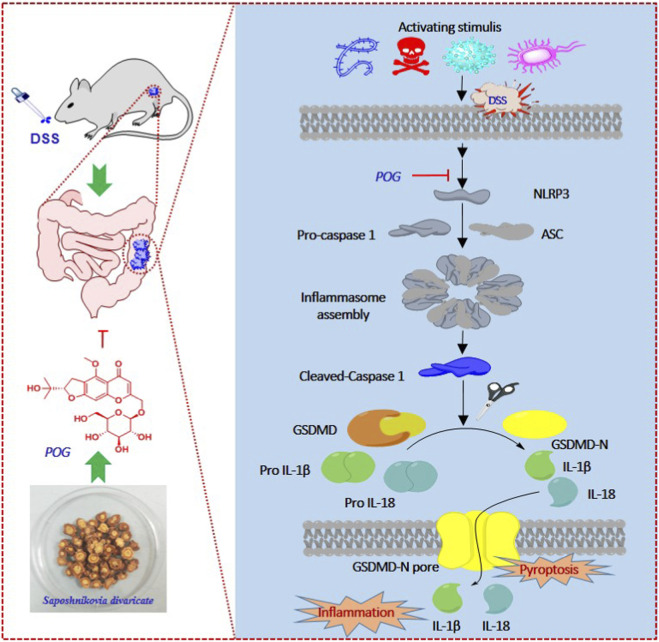
Mechanism of action of POG in the treatment of UC.

Analysis of the experimental results (Figure 6) demonstrates that POG administration significantly downregulates protein expression levels of NLRP3, gasdermin D-N (GSDMD-N), cleaved IL-1β and cleaved caspase-1, indicating that POG exerts its anti-inflammatory effects by suppressing the NLRP3 inflammasome activation. This suppression prevents the proteolytic activation of pro-caspase-1 and GSDMD, thereby reducing GSDMD-N-mediated pore formation on the cell membrane and subsequent IL-1β release. Consequently, this blockade attenuates downstream inflammatory responses, mitigates damage to the colonic barrier system, and inhibits disease progression. These findings were corroborated by ELISA data showing decreased levels of cytokines IL-1β, TNF-α, IL-6, and IL-18 in treatment groups, as well as histopathological assessments confirming tissue integrity. Collectively, the experimental evidence indicates that POG alleviates UC by inhibiting NLRP3 inflammasome activity, suppressing inflammatory responses and ameliorating disease symptoms (Figure 7).

Importantly, although our animal experiments provide preliminary evidence for the anti-ulcerative colitis mechanism of POG, mechanistic validation at the cellular level is still essential. Specifically, *in vitro* studies using macrophage models (e.g., LPS-stimulated RAW 264.7 cells) are needed to directly evaluate inflammatory cytokine responses and key pyroptosis/inflammasome-related proteins, including NLRP3, ASC, caspase-1, GSDMD, and IL-1β processing. These experiments were not included in the present study, which represents a limitation. Another limitation of this study is that inflammatory cytokines were measured only in serum and not in colon tissue homogenates nor assessed by qPCR to evaluate local inflammation. Therefore, further cell-based investigations are required to confirm the direct molecular targets of POG and to more fully elucidate its anti-inflammatory mechanism. Overall, these findings support that POG alleviates experimental UC by targeting the NLRP3/caspase-1/GSDMD axis and protecting intestinal barrier function. This study provides mechanistic evidence for POG as a promising candidate for UC treatment and offers a basis for future translational and therapeutic investigations.

A limitation of this study is that inflammatory cytokines were measured only in serum and not in colon tissue homogenates nor assessed by qPCR to evaluate local inflammation.

## 5 Conclusions

This study elucidates the molecular mechanism by which POG enhances intestinal barrier function through the regulation of the NLRP3 inflammasome/GSDMD pathway, providing new experimental evidence for the use of active components of traditional Chinese medicine for treating UC. These findings not only expand the understanding of the pharmacological actions of traditional Chinese medicine but also identify potential candidate molecules for the development of novel anti-inflammatory therapies. Future research should further investigate the translational and clinical potential of POG to offer safer and more effective treatment options for UC.

## Data Availability

The original contributions presented in the study are included in the article/supplementary material, further inquiries can be directed to the corresponding authors.
